# Supplementary material for: The fisheries governance tool: A practical and accessible approach to evaluating management systems
Source: PLoS One. 2021 Jul 1;16(7):e0253775. doi: 10.1371/journal.pone.0253775 (PMC8248635; doi:10.1371/journal.pone.0253775)
Supplement: S1 Table — (DOCX) [file pone.0253775.s001.docx]

S1 Table 1. Standards of evidence and examples for assessing whether or not a Measure is met under Component 1: Policy.

| **Measure Met?** | **EVIDENCE** | **EXAMPLES** |
| --- | --- | --- |
| **Yes** | Documentation from within the last two years that a policy exists, published or unamended, by the relevant authority. Details related to a given measure are explicit or implicit, as appropriate in the policy. | Constitution, statute, decree, policy, regulation, annual report, government testimony, government websites for fisheries administrations. Credible summary report and publications can also provide evidence of policy with appropriate citations. The assessor should be able to verify that in fact that country does meet the measure. |
| **In Part** | Documentation that a policy is under consideration, an existing policy meets the measure part way, or that an existing policy was amended in a way that makes it less effective to meet the measure.  Documentation that a policy exists, such as a news report or public announcement, but has not been published by the decision-making authority. | The evidence required for ‘Yes’ or ‘In Part’ apply at both scoring levels with the understanding of whether a measure is completely met or requires additional capacity to meet.  Policy documents, peer reviewed literature, court cases, foundation reports, expert interviews, government websites, official speeches, media articles that mention or report information related to the measure. The assessor should be able to verify that the measure is partially met, but there is a gap with respect to completely meeting the measure. |
| **No** | No policy document or legal instrument states a requirement for, or existence of, the measure. The policy or related document may provide evidence for both meeting and not meeting measures, such as defining objectives. | The measure is not addressed in published information; revised statute or regulation no longer contains the measure; relevant authority states the measure is not required. In scoring a 'No', there is definitive evidence that the country does not support the measure. The assessor should be able to verify that in fact the country doesn't meet the measure. |
| **Not Evaluated** | The measure cannot be evaluated because data are unavailable. | Unpublished, inaccessible, not collected. In scoring 'Not Evaluated', no information was found in support or lack of support for the measure. Searches or communications with experts revealed that there is no information available on this measure. Of course, this can be the most challenging to score, as knowing when to end a search for information and definitively score ‘Not Evaluated’ is challenging. |
