# Supplementary material for: The fisheries governance tool: A practical and accessible approach to evaluating management systems
Source: PLoS One. 2021 Jul 1;16(7):e0253775. doi: 10.1371/journal.pone.0253775 (PMC8248635; doi:10.1371/journal.pone.0253775)
Supplement: S2 Table — (DOCX) [file pone.0253775.s002.docx]

S2 Table 2. Standards of evidence and examples for assessing whether or not a Measure is met under Component 2: Capacity.

| **Measure Met?** | **EVIDENCE** | **EXAMPLES** |
| --- | --- | --- |
| **Yes** | Capacity and resources are present in budget allocations, human resources, and competency in science, Monitoring/Compliance/ Surveillance, and infrastructure. | To meet these measures, there should be evidence of annual budget allocations; strategic plans, organizational diagrams, dedicated offices and divisions, and demonstration through annual reports, scientific publications, stock assessments, monitoring and compliance reports, audits, investigative reports by third parties or government oversight bodies. These may be demonstrated on government websites, in summary papers, publications and related credible references. The assessor should be able to verify that in fact that country does meet the measure. |
| **In Part** | Documentation that the fishery management authority has resources but may be insufficient to achieve policy goals and objectives, unreliable or inconsistent year to year; documentation that additional resources are required to meet management obligations. | The evidence required for ‘Yes’ or ‘In Part’ apply at both scoring levels with the understanding of whether a measure is completely met or requires additional capacity to meet.  Peer reviewed literature, organization charts, budget request justifications, expert interviews, government websites, official speeches, media articles that mention or report shortfalls or problems. The assessor should be able to verify that the measure is partially met, but there is a gap with respect to completely meeting the measure. |
| **No** | The absence of budget or planning document, or administrative policy document that states a requirement for or existence of the capacity measures. | The measure is not addressed in published information (as far as can be concluded); revised budget or agency organization chart no longer contains the measure; relevant authority states the measure is not required. There may be reference in reports that capacity is needed in certain areas, but not provided. In scoring a 'No', there is definitive evidence that the country does not support the measure. The assessor should be able to verify that in fact the country doesn't meet the measure. |
| **Not Evaluated** | The measure cannot be evaluated because data are unavailable. | Unpublished, inaccessible, not collected.  In scoring 'Not Evaluated', no information was found in support or lack of support for the measure. Searches or communications with experts revealed that there is no information available on this measure. Of course, this can be the most challenging to score, as knowing when to end a search for information and definitively score ‘Not Evaluated’ is challenging. |
