# Supplementary material for: The fisheries governance tool: A practical and accessible approach to evaluating management systems
Source: PLoS One. 2021 Jul 1;16(7):e0253775. doi: 10.1371/journal.pone.0253775 (PMC8248635; doi:10.1371/journal.pone.0253775)
Supplement: S3 Table — (DOCX) [file pone.0253775.s003.docx]

S3 Table 3. Standards of evidence and examples for assessing whether or not a Measure is met under Component 3: Fisheries Performance.

| **Measure Met?** | **EVIDENCE** | **EXAMPLES** |
| --- | --- | --- |
| **Yes** | The fishery-specific management system has demonstrated the existence of the measure (e.g. where requiring specific management tools or data collections) and fishery performance outcomes at the relevant scale (where evidence to support demonstration of outcomes does not exist, but the requirement for the measure is present, score the fishery 'In-Part' for the given measure). | Law or regulation, fishery management plans, annual or biennial stock assessments and status reports, plan amendments, rebuilding plans, scientific and technical committees, science agencies, published research, external credible reports from ratings and certifications organizations, Vessel Monitoring System or similar Monitoring/Compliance/Surveillance requirements. The assessor should be able to verify that in fact that country does meet the measure. |
| **In Part** | Fisheries management plan, strategy, detailed regulations, or other management document describes the measure, but does not require it—or does not enforce it; or the measure is an objective, but attainment of the objective cannot be documented. | The evidence required for ‘Yes’ or ‘In Part’ apply at both scoring levels with the understanding of whether a measure is completely met or requires additional capacity to meet.  Credible summary reports and meeting outputs, rebuilding plans, stock assessments, discard data, compliance reviews, scientific reviews, scientific and technical committee reports that mention or report shortfalls or problems. The assessor should be able to verify that the measure is partially met, but there is a gap with respect to completely meeting the measure. |
| **No** | There is documentation that the fishery management actions do not include a conservation and management measure. | The measure is not addressed in published information; revised plan, strategy or regulation no longer contains the measure; relevant authority states the measure is not required. In scoring a 'No', there is definitive evidence that the country does not support the measure. The assessor should be able to verify that in fact the country doesn't meet the measure. |
| **Not Evaluated** | The measure cannot be evaluated because data are unavailable, or the assessor does not know the answer and could not identify supporting evidence. | Unpublished, inaccessible, not collected. A measure related, for example, to shared stocks, transboundary stocks, or distant water fleets may not be applicable at the fishery level. In scoring 'Not Evaluated', no information was found in support or lack of support for the measure. Searches or communications with experts revealed that there is no information available on this measure. Of course, this can be the most challenging to score, as knowing when to end a search for information and definitively score ‘Not Evaluated’ is challenging. |
| **Not Applicable (NA)*** | This score only applies to Component 3. | This may happen where certain measures do not apply to the characteristics of a fishery being assessed. For example, if the fishery is not a transboundary stock, there would not be the need for fishery-specific management to participate in international agreements, and therefore the measure would not be scored, and the reason would not be for lack of information. However, for example, a nation scored under Components 1 and 2 would still be required to adhere to international agreements on transboundary stock management. |
| **We theorize that in the policy and capacity components, statements about how they will sustain the fisheries resources will be scored as ‘Yes’ or ‘No’ and should not be regarded as ‘Not Applicable’.* | | |
