# Supplementary material for: The fisheries governance tool: A practical and accessible approach to evaluating management systems
Source: PLoS One. 2021 Jul 1;16(7):e0253775. doi: 10.1371/journal.pone.0253775 (PMC8248635; doi:10.1371/journal.pone.0253775)
Supplement: Attachment — Submitted filename: Response to Reviewers.docx [file pone.0253775.s004.docx]

*8950 Martin Luther King Jr St N, Suite 202*

*St. Petersburg, FL 33702 USA*

*Tel: (727) 563-9070*

*Fax: (727) 563-0207*

*Email: MRAG.Americas@mragamericas.com*

*President: Andrew A. Rosenberg, Ph. D.*

Friday, April 30, 2021

Dear PLOS One Editorial Board,

We are pleased to submit the revised manuscript *The Fisheries Governance Tool: A Practical and Accessible Approach to Evaluating Management Systems* for your consideration.

In the table below we list the points raised by the review and identify the changes we have made in response. We also include an updated Funding Statement and Competing Interests Statement below.

We look forward to your review of our revised manuscript.

Sincerely,

Graeme Parkes

(Graeme.parkes@mragamericas.com)

| **Item** | **Response (line numbers refer to the final version without tracked changes)** |
| --- | --- |
| Please provide an amended Funding Statement | We have provided an amended Funding Statement, including a statement regarding the Role of Funders in our study. |
| Please provide an amended Competing Interests Statement | We have provided Competing Interests Statement. |
| Please include captions for your Supporting Information files at the end of your manuscript, and update any in-text citations to match accordingly. Please see our Supporting Information guidelines for more information: <http://journals.plos.org/plosone/s/supporting-information>. | Captions for our Supporting Information files have been added at the end of our manuscript. |
| **Comments to the Author, abstracted from the full text.** |  |
| Provide a short outline of the paper | Added text at the end of the Introduction– lines 81 to 86 |
| The attention to WFF in the introduction is not fitting with the nature of an academic publication. WFF's role should not be understated, but the relevance of this tool goes beyond its use by WFF (as the authors rightly state). | Edited the Introduction to focus more on the FGT than WFF, and also the motivation for preparing the manuscript for publication (lines 75-80). |
| Put that broader relevance/usability forward in the introduction. | Edits to the Introduction in response to the previous comments also address this comment, e.g. lines 75-80 and 81-86. Also there is text in the new section 2 (previously 1.1/1.2 that better explains the relevance of the tool in the context of other tools . |
| Better explain the tool:  Elaborate more on the underpinning of their tool, for example it would be helpful if they would clarify what theoretical notions are key to their understanding of governance.  The three components "policy, capacity and performance" are relevant, yet it is presented being common sense, rather than explained and substantiated. There is perhaps no need to define policy, but what is "capacity" or "performance" to the authors? Even some more attention to the choice of underpinning the tool by the "triple bottom line" would help understanding its position. | The explanation of three components of policy, capacity and performance is expanded in new text in section 3.1 (Outline of the FGT) (lines 194-212). |
| The introduction now concludes with a claim about the FGT, but it is unclear whether this is what we will see evidenced in the paper. From my reading, I don't think the authors show "the FGT as a means of consistently assessing a country's progress over time" and how the FGT provides a road map. | This claim is deleted. While true, the claim relies on the consistent use of the tool over time, and is not a characteristic of the tool itself. |
| Absence of connection to the academic literature :  The paper would benefit from a more specific linking between section 1.1 and 1.2. Doing so, the authors could balance in 1.1 between what is context and what has been taken from the review to develop the FGT. Also, a more specific comparison would clarify the specific characteristics of the FGT, such as why the FGT is adaptable in scale, while the FPIs are not (because one could argue that, in principle, they are too).  Although Anderson et al's FPIs are mentioned, there is little contrasting so to understand what has been learnt/taken from Anderson et al, and how the FGT is different. In reading, I felt it is mentioned but the how is not well-explained. | Sections 1.1 and 1.2 have been amalgamated in a new section 2. The new section presents a more evenly weighted description of existing evaluation mechanisms and concludes with a more clear explanation of the design criteria for the FGT leading to the decision to build a novel tool. |
| • In section 2.3.2. scoring, the authors mention that the four scales (basic; adequate; good; better) for each measure are scored at an incremental rate which is quite sensible (even though a bit arbitrary). However, it is not clear whether such differential weighting/scoring scheme is applied across measures within the same component, and across components. If not, how it is expected that different measures contribute with same effectiveness towards the sustainability/efficiency of a fishery? | New text has been added to more fully explain the scoring and it’s limitations in this first iteration of the Tool (lines 366-397) |
| • Even though the authors discuss about data-deficient fisheries at a few places, they may elaborate on how the Tool can circumvent the problems of small-scale fisheries where adequate systems are not in place to systematically document different aspects of the fishery, as the Tool demands. Here, I would like to refer the specific case of certain island ecosystems where the ecosystem is pristine and the inhabitants take utmost care in maintaining sustainable fishing practices over centuries based on traditional management approaches, but they do not have sufficient data / documentations in place. | We have added new text into Section 4 Discussion regarding use of the Tool with small scale and data deficient fisheries (lines 503-524). |
| • The authors may also elaborate on how non-state legal systems/co-management systems are taken into account in the Tool under different components / indicators / measures. | The new text added into Section 4 Discussion also addresses this point (lines 503-524). |
| • Whether the Tool has the potential to be utilized for developing independent eco-certification schemes in the future? – This aspect may be discussed in the concluding section. | We have declined to comment on this specifically, believing it would be premature. The Tool could be used in a variety of ways, some of which have not been identified, and/or thought of as yet, and may only become apparent after a period of use in a variety of circumstances. The Tool is specifically set up without an externally set standard or benchmark, leaving it up to the user to set their own goals. The FGT is not intended to be used as a certification scheme, but neither do we feel we should explicitly state this in the manuscript, given the debate such an explicit “denial” might provoke. We consider such a debate would detract from the central purpose of the Tool at this early stage. Nevertheless, we would be potentially interested to revisit this in the future. |

**Funding Statement and the Role of Funders.**

The authors did not receive “grants” for this work. Three authors (KS, MS, CR) were employees of the Walton Family Foundation. Three authors (JS, GP, BT) were employees of MRAG Americas, Inc. (MRAG Americas). One author (SI) was a contractor to MRAG Americas. The “funding” structure was a contract between a foundation and a consulting firm. Commercial affiliations, and the role of funders role are described below.

The work, of which the submitted article was a minor part, was supported by the [Walton Family Foundation](https://www.waltonfamilyfoundation.org/) through a contract to [MRAG Americas](https://www.mragamericas.com/) Fisheries Technical Division. MRAG Americas is a private consulting and auditing company focused on activities that support the conservation of marine and freshwater ecosystems. MRAG Americas’ Fisheries Technical Division conducts analyses of fisheries policy, management, and performance for a variety of clients including environmental foundations, non-government environmental organizations, state, federal and international management agencies, fishery management councils, and industry groups.

The contract to create a rubric for scoring national fisheries management policy was awarded to MRAG Americas by the Walton Family Foundation Strategy, Learning and Evaluation Division, originally in 2016 with extension years to 2020 (contract #s C2016-403, C2017-1633, C2019-2857 and 00102041). A subcontract was awarded to Iudicello by MRAG Americas in 2016 with extension years to 2020. The scope of work included research on existing performance assessment schemes, identification of best practices, development of the analytic framework for a scoring tool, workshopping and testing the tool with outside experts, and creating an online, no-cost, downloadable tool. In addition, the deliverables included two rounds of completed scoring of five countries, and submission of an article about the development and application of the tool to a peer reviewed journal. All deliverables were completed. The contract ended in December, 2020.

The funder provided support in the form of salaries for authors CR, MS and KS. The funder provided terms of reference for the study, as outlined in the manuscript, and participated in the selection of case study countries, but did not have any additional role in the data collection and analysis, decision to publish, or preparation of the manuscript. The specific roles of these authors are articulated in the author contributions section. At the onset of the contract, co-authors CR and MS were on the staff of Walton’s Strategy, Learning and Evaluation division. In 2018, co-author KS joined the Walton staff.

At the onset of the contract, corresponding author GP, and co-authors JS and RT were employees of MRAG Americas. Billable hours related to the project were charged to the Walton Family Foundation contract. The author contributions section of the submission describes the roles of each of these co-authors.

Co-author SI is an independent researcher, writer and consultant, and sole proprietor of [Iudicello Environmental Consulting](https://www.linkedin.com/in/suzanne-iudicello-165468b). A sub-contract with MRAG Americas from the onset of the Walton project was one of more than 10 separate clients/projects during the period 2016-2020. Billable hours related to the project were charged to the MRAG Americas sub-contract. The author contributions section of the submission describes the role of this co-author.

**Competing Interests Statement**

The authors have no competing interests. The work conducted by MRAG Americas and by Iudicello did not interfere with, nor could be perceived as interfering with the development of the Fisheries Governance Tool or the authorship of the article describing the project the full and objective presentation, peer review, editorial decision-making, or publication of research or non-research articles submitted to one of the journals.

The corresponding author confirms that affiliation with MRAG Americas and Iudicello Environmental Consulting does not alter our adherence to PLOS ONE policies on sharing data and materials.
